# Professional Empiricism

**Published:** 1854-02

**Authors:** Geo. W. Hall

**Affiliations:** Carthage, Ill.


### *Professional Empiricism.*

BY GEO. W. HALL, M. D. CARTHAGE, ILL.

As a great deal has recently been written on quackery of one kind or other, and thinking that attention might be profitably directed to empiricism as it exists in the profession, I will mention a few things which I deem to be wrong. Most writers refer to quackery out of the profession, and speak of those who prey on the public and make no claims to legitimate medicine. This is well enough, and such imposters deserve all they get and more, but I insist that writing and speaking against *pretenders* is not the most effectual way to put them down.

Those communities of christians flourish best where each member attends strictly to the practice of religion—obeys all requirements of duty and does not abuse others who think and act differently.

It is just so in Medicine, and if physicians will attend faithfully to their proper business—the study of man's complex conformation—his physiology—the many diseases to which he is incident—the proper means to be used in alleviating his sufferings—and the most rational system of prophylais possible for human wisdom to devise and institute—the science, that most noble and exalted of all others, will become as a "city set upon a hill" shedding light and knowledge on all around, and rendering man's pathway from the "cradle to the tomb" radiant with health and prosperity.

This is, I think, the proper and only true business of the physician, and if every one were to do so, mere pretenders, and those imposters would be compelled of *necessity* to leave the field to its rightful owners. But instead of this, many to whom the people look as the embodiment of professional knowledge and integrity, declaim loudly against the *outsiders*, to the neglect of that most important and essential requisite, self culture.

The physician who will attend assiduously to the discharge of the manifold duties of his profession, will always have his mind and attention usefully occupied, and will not *stoop* to notice every charlatan he must necessarily often meet. But there are those who pay a total disregard to most points of professional honor, and sometimes decency, in order to gain practice, and the esteem of the conceited, and foolish among the community.

There are others in the habit of so prostituting the profession, as to recommend the use of patent medicines, where they find their customers in favor of their use. I know an individual possessing a fair share of patronage, who often permits his patients to use "Davis' Pain Killer," both internally and externally, and other similar nostrums, along with his remedies. Can the public have full confidence in medicine, when those regarded as regular physicians act thus? How much better it would be for that individual—his patrons—and the science—which he thus dishonors—if his influence were placed in opposition to such nostrums. Such a man does the profession more harm, and by his influence assists more in bringing it into disrepute than a dozen "claudentine physicians," and until it is purged of such we may look for impostors of various kinds, to hold an enviable place in public confidence, and favor.

There are others, who cater to the morbid appetites of the vulgar in other ways equally disreputable, and opposite to professional etiquette.

I allude to the custom of slandering competitors—of cringing to the wealthy for their influence—of undercharging—of adopting the creed of some church merely for "material aid" and many other things known to all, and known to be an outrage on the fair name of the profession.

Those who dishonor their professsion, dishonor themselves—lose everything and gain nothing.

Others sacrifice their standing among physicians at the shrine of mammon, but what will a few hundred dollars be worth when gained at such a sacrifice? To "live a life of duty done," should be the aim of every physician—the goal of his ambition, and it is the only sure way of having an unblemished character while living, and leaving an untarnished name to posterity.

